# 3D Printing of Thermoresponsive Hydrogel Laden with an Antimicrobial Agent towards Wound Healing Applications

**DOI:** 10.3390/bioengineering8060079

**Published:** 2021-06-08

**Authors:** Martyna Nizioł, Justyna Paleczny, Adam Junka, Amin Shavandi, Anna Dawiec-Liśniewska, Daria Podstawczyk

**Affiliations:** 1 Department of Process Engineering and Technology of Polymer and Carbon Materials, Faculty of Chemistry, Wroclaw University of Science and Technology, Norwida 4/6, 50-373 Wroclaw, Poland; martyna.niziol@pwr.edu.pl; 2 Department of Pharmaceutical Microbiology and Parasitology, Wroclaw Medical University, 50-556 Wroclaw, Poland; justyna.paleczny@student.umed.wroc.pl (J.P.); adam.junka@umed.wroc.pl (A.J.); 3 BioMatter Research Unit-Biomass and Biomaterials (3BIO-BioMatter), Université Libre de Bruxelles, Avenue F.D. Roosevelt, 50, CP 165/61, 1050 Brussels, Belgium; amin.shavandi@ulb.be; 4 Department of Advanced Material Technology, Faculty of Chemistry, Wroclaw University of Science and Technology, M. Smoluchowskiego 25, 50-372 Wroclaw, Poland; anna.dawiec@pwr.edu.pl

**Keywords:** stimuli-responsive, wound patch, biocompatible, additive manufacturing, printability

## Abstract

Thermoresponsive hydrogel-based wound dressings with an incorporated antimicrobial agent can be fabricated employing 3D printing technology. A novel printable ink containing poly(N-isopropylacrylamide) (PNIPAAm) precursors, sodium alginate (ALG), methylcellulose (MC) that is laden with a mixture of octenidine dihydrochloride and 2-phenoxyethanol (Octenisept^®^, OCT) possess accurate printability and shape fidelity. This study also provides the protocol of ink’s use for the 3D printing of hydrogel scaffolds. The hydrogel’s physicochemical properties and drug release profiles from the hydrogel specimens to the external solution have been determined at two temperatures (20 and 37 °C). The release test showed a sustained OCT delivery into ultrapure water and the PBS solution. The temperature-responsive hydrogel exhibited antimicrobial activity against *Staphylococcus aureus*, *Candida albicans*, and *Pseudomonas aeruginosa* and demonstrated non-cytotoxicity towards fibroblasts. The thermoresponsive behavior along with biocompatibility, antimicrobial activity, and controlled drug release make this hydrogel a promising class of materials for wound dressing applications.

## 1. Introduction

Tissue engineering for chronic wound healing is a multidisciplinary approach that combines material science, life science and medical sciences such as anatomy or physiology; additionally, computer modeling is used to design and produce effective, biomimetic artificial materials for in situ regeneration of nonhealing wounds [1]. Due to the complex nature of human skin tissue and its anisotropic properties, the creation of functional, biocompatible artificial tissue/skin substitutes with suitable mechanical and chemical resistance and appropriate composition of proteins, cells, and other active agents which should be additionally aligned in a very specific order, is still very challenging. Non-healing injuries, such as diabetic wounds, pressure sores, or venous ulcers represent a crucial healthcare problem because in most clinical cases they gradually turn to be chronic due to ineffective healing procedures. Therefore, chronic wound healing is an unmet challenge among the world’s pharmacology industries and medical society [2]. Available therapeutic approaches to non-healing injuries used in the clinics —such as diabetic wounds, pressure sores, burns, or venous ulcers—are still insufficient, time-consuming, and present restricted customization. In response to this, nowadays, personalizable bioengineered wound dressings and bio-fabrication techniques are being extensively developed. New research in chronic wound healing presents an innovative therapeutic strategy by using three-dimensional (3-D) hydrogel-based materials with superior therapeutic potential [3,4]. Hydrogel-based chronic wound dressings exhibit multifunctional properties, such as biodegradability, biocompatibility, sustained diffusion of bioactive molecules, high cell/drug embedding rate, pore structure, and appropriate mechanical strength [5]. Additionally, modern hydrogel patches have been engineered to absorb large amounts of wound exudate while maintaining an appropriate amount of moisture at the interface between a healing wound and an applied 3-D dressing [6]. These three-dimensional hydrogel-based networks can be directly placed/inject into the target wounds [3,7] or created by adopting novel approaches, such as 3-D printing or electrospinning [8,9]. Nowadays, these innovative techniques have been attracting increasing attention as alternative 3-D scaffold-forming approaches for the enhancement of chronic wound therapies. Electrospinning is an effective method to produce micro- and nano-fiber three-dimensional networks based on biocompatible polymers, with the proper porosity and a high degree of morphological resemblances to ECM. Nevertheless, natural polymers are often challenging for this approach [10]. In comparison with conventional methods, 3-D printing allows for the fabrication of flexible, repeatable, personalized, and anatomically fitting structures with design morphology and composition as well as with high complexity [11,12]. Advances in 3D printing technology have enabled 3D fabricating of functional scaffolds for tissues from both natural and synthetic biocompatible materials, cells, and cell-support materials. This technology is now widely used in regenerative medicine, tissue engineering, and implantology [13,14]. It operates in the following fashion. A pre-designed model is converted to a real object by dispensing layers of the biomaterial or bioink laden with biologically active molecules or cells in an extrusion-based method to produce a pre-designed model of the desired shape [14]. The deposition of the material on the substrate is strictly controlled [15]. It is used for the fabrication of tissue-mimicking and smart hydrogel inks and their non-invasive application in wound healing dressings [2,16,17,18,19,20].

Functionalization of hydrogel networks with bioactive agents such as immunomodulating molecules [21], growth factors [22], and antibacterial agents [23] is a promising approach that can be used for fostering the wound repair process by the slow and sustained release of biomolecules at the wound bed. The selection of topical active agents is significant for the preparation of new multifunctional chronic wound healing materials. Octenidine dihydrochloride (OCT) is a widely used antiseptic that promotes skin wound healing and improves scar quality after burns and surgical interventions [24]. It is an alkanediylbis[pyridine] germicidal agent with structural similarity to chlorhexidine and benzalkonium chloride [25,26]. It exhibits stability upon exposure to light and in a wide pH range of 1.6–12.2. OCT shows high microbiocidal activity against a vast spectrum of common human pathogens. Hydrogels containing OCT have been already developed to create a moist wound environment and to protect the wound from exposure to microorganisms under occlusive dressings [26,27,28]. Bernardelli de Mattos et al. developed hydrogels based on bacterial nanocellulose (BNC) laden with OCT as an excellent carrier for creating on-demand antiseptic wound dressings. It ensured a continuous release of antiseptic and showed high antimicrobial efficacy against *Staphylococcus aureus* [28]. Junka et al. developed not only OCT-saturated nanocellulose-based dressings but also a simple method for evaluating their anti-biofilm activity against common wound pathogens (*Staphylococcus aureus* and *Pseudomonas aeruginosa*) [29]. Cellulose-based materials, such as bacterial nanocellulose, have been the common components of soft-gels that are particularly useful for wound care products. The water-retaining capability, good tunability, and highly porous structure make cellulose an excellent material for wound recovery [30,31]. However, only a few papers have reported methylcellulose (MC) as a building block of wound dressings. MC hydrogel can be easily injected in situ and as easily integrates with the living tissue filling a wound preventing microbial infection [32,33]. In 3D printing, on the other hand, MC has acted as an ink’s viscosity modifier or inks’ sacrificial component [34,35,36]. In this study, MC was analyzed for its dual role of improving the rheological properties of the hydrogel precursor and enhancing the hydrogel microbiocidal efficacy.

In the last two decades, thermo-reversible hydrogels with temperature-triggered sol-gel transition capability have received significant attention for use in drug delivery systems. Among temperature-stimulated polymers, poly(N-isopropylacrylamide) (PNIPAAm) has been intensively investigated for tissue engineering and wound dressing materials [37]. Because of a low (32 °C) critical solution temperature (LCST), PNIPAAm can form a gel at the body temperature. PNIPAAm’s LCST can be elevated to 37 °C through co-polymerization expanding its applicability in healthcare. Hybrid PNIPAAm-based networks have demonstrated a high potential as injectable wound closures [38,39,40,41]. In a recent paper, Blacklow et al. developed tough adhesive hydrogel with high stretchability and strong tissue adhesion based on PNIPAAm and alginate with silver nanoparticles as an antimicrobial agent [2]. The dressings adhered strongly to porcine and wounded rodent skin and generated sufficient temperature-activated wound contraction to promote healing. The authors were inspired by negative-pressure wound therapies and embryonic wound healing, where a gap in the embryonic epidermis is closed by contraction of a rapidly assembled actin purse-string [42]. A similar approach was presented by Li et al., who reported a biomechanically active and biochemically functional hydrogel based on the quaternized chitosan (QCS), polydopamine-coated graphene oxide (rGO-PDA), and PNIPAAm as a wound dressing possessing multiple functions and properties such as self-healing, temperature-dependent drug release, anti-infection, antioxidation, and conductivity [40]. Adapting PNIPAAm to serving as ink for direct printing is an innovative bioengineering strategy toward wound healing applications that has not been reported in the scientific literature.

By combining excellent properties of MC, PNIPAAm, and alginate (ALG) with extrusion-based direct printing technology, we developed a new 3D-printable hydrogel. The hydrogel contains an interpenetrating network of dually crosslinked PNIPAAm and alginate along with MC as the viscosity modifier. This concept arose from our previous study which reported 4D thermoinks composed of Laponite^®^, an interpenetrating network of PNIPAAm and ALG for direct printing of 3D honeycomb-patterned hydrogel discs self-rolling into tubes [37]. In this study, poly(ethylene glycol) diacrylate (PEGDA) was introduced as a hydrophilic co-monomer and a long-chain crosslinker of PNIPAAm to enhance the biocompatibility of the PNIPAAm network. A hybrid PNIPAAm-alginate network was first engineered for shear-thinning behavior, high printability, and shape fidelity by optimizing the inks’ composition and direct printing parameters. The hydrogel was further loaded with Octenisept^®^ rendering the material antimicrobial. The printable material demonstrated multifunctionality: (1) temperature-induced shrinkage to potentially accelerate wound contraction, (2) biocompatibility, and (3) antimicrobial activity.

## 2. Materials and Methods

### 2.1. Materials for the Ink

N-isopropylacrylamide (NIPAAm, purity 97%), *N,N*′-methylenebis(acrylamide) (MBA, purity 99%), 2-hydroxy-4′-(2-hydroxyethoxy)-2-methylpropiophenone (I2959, purity 98%, CAS #106797-53-9), poly(ethylene glycol) diacrylate (PEGDA, Mn 575), sodium alginate (ALG, Mw ≈ 120,000–190,000; M/G ratio 1.56), phosphate-buffered saline (PBS), calcium chloride dihydrate, MC (4000 cP) and inhibitor remover were purchased from Sigma-Aldrich (Saint Louis, MO, USA) and used as received. Octenisept^®^ containing 0.1% *w*/*w* of octenidine dihydrochloride and 2%*w*/*w* of 2-phenoxyethanol was acquired from the local drugstore. Ultrapure water (pH~7.0) was filtered through a 0.220 µm PES membrane filter, sonicated, and purged with nitrogen to remove the oxygen dissolved.

### 2.2. Preparation of the Ink and 3D Printing

The protocol of the ink preparation was based on the previous studies [37,43]. A dark glass vial filled with ultrapure water (V = 10 mL) was placed on a magnetic stirrer at 400 rpm. Subsequently, 1.13 g of NIPAAm was added, followed by 31 mg of MBA and 88 mg of I2959. While the mixture was being stirred until homogeneity was attained, 0.21 mL of PEGDA was passed through the column containing the inhibitor remover and added to the vial. The stirring speed was raised to 1500 rpm and 0.82 g of sodium alginate was slowly admixed to the solution. After 30 min, the hydrogel precursor was heated up to 80 °C, and 0.82 g of MC–whose amount corresponded to ALG:MC mass ratio of 1:1–was poured in until a uniform suspension was obtained. Finally, the ink was cooled in an ultrasonic-assisted water bath for 30 min. The hydrogel precursor (ink) was carefully poured into a disc-like plastic mold with a diameter of 8 mm and height of 5 mm, followed by curing under the UV light (10 mW cm^−2^, 365 nm) for 5 min to fabricate cylindrical specimens for characterization. The specimens were transferred to calcium chloride solution (0.5 M) for 24 h for ALG crosslinking.

Before printing, the ink was transferred into a 3 mL UV-shielded syringes, stored overnight at ~8 °C, and then centrifuged at 5800 rpm for 10–15 min at room temperature to remove air bubbles. A temperature-controlled pneumatic-based extrusion printer (BioX, Cellink, Sweden) carried out the 3D printing. The hydrogel precursor was pneumatically extruded through the 27G nozzle (200 µm) at a pressure of 150 kPa and speed of 3–5 mm/s and deposited onto a temperature-controlled stage along the designed path kept at room temperature. Completed 3D scaffolds were cured under UV light for 5 min and then immersed in 0.5 M calcium chloride solution for 24 h for Ca^2+^ cross-linking. Afterward, the specimens and scaffolds were washed several times with 96% ethanol and ultrapure water to remove unreacted species and then stored in ultrapure water. The washing procedure includes at least five cycles of alternating water/ethanol incubation for 24 h followed by 5 repetitions of ultrapure water rinsing of the samples.

### 2.3. Characterization of the Ink and Hydrogel

#### 2.3.1. Rheological Measurements

Ink’s rheology was characterized via testing on a parallel-plate rotational rheometer Physica MCR 501 (Anton Paar, Graz, Austria) with a 40 mm diameter plate and 1 mm gap between plates. The tests were conducted via a fixed frequency of 1 Hz within a strain range of 0.01–100% and a frequency sweep of 0.01 Hz to 100 Hz at a strain of 0.15%. All experiments were performed at a temperature of 25 ± 0.1 °C.

#### 2.3.2. Compression Tests

Cylindrical specimens were subjected to the static uniaxial compression test performed using the MULTITEST1-i machine (Mecmesin, Slinfold, West Sussex, UK). The compression tests were carried out at a speed of 0.5 mm/min until the first instance of the reduction of the compressive force signaling the appearance of permanent deformation of the test sample. Based on the registered values of force and displacement, stress–strain curves were determined, which allowed for determining the value of compressive strength and Young’s modulus.

#### 2.3.3. ATR

Attenuated total reflectance-Fourier transform infrared (ATR-FTIR) spectra of the hydrogels and their components were recorded on a Bruker Vertex 70 instrument (Bruker, Billerica, MA, USA) equipped with an ATR accessory within a scan range of 4000–400 cm^−1^ and a resolution of 1 cm^−1^.

#### 2.3.4. Swelling Measurements

Before the swelling measurements, the samples were dried in a vacuum oven at 50 °C. Dry hydrogel cylindrical specimens were first weighed and then immersed in 10 mL of ultrapure water in sealed glass flasks at room temperature for 24 h. Subsequently, the samples were incubated in 20 and 37 °C maintained with a cryostat (Julabo FB50, JULABO GmbH, Seelbach, Germany). The hydrogels were kept for 5 min to 24 h at each temperature. The samples were removed from the flask, and the excessive amount of the solvent was wiped off with a paper towel, and then weighed (m_wet_ [g]). The swelling ratio (Swelling (%)) was calculated from the following Equation (1).

(1) $$\mathrm{Swelling}=\frac{m_{\mathrm{wet}}-m_{\mathrm{dry}}}{m_{\mathrm{dry}}}\times 100\%$$

where m_dry_ [g] is a mass of dry hydrogel. Additionally, the specimen diameter and height at 20 and 37 °C were measured.

For the investigation of the reversibility of swelling-shrinkage behavior, the samples were cyclically immersed in heated (37 °C) and cooled (20 °C) water for 120 min. The sample mass and diameter were measured at each heating-cooling cycle and swelling was calculated.

#### 2.3.5. Scanning Electron Microscopy

Before imaging, the hydrated samples were placed on a cryogenic stage, immersed in liquid ethane, followed by lyophilization. The cross-section morphology of the hydrogels was analyzed with a scanning electron microscope (SEM), Quanta 250 FEG 1 (FEI, currently ThermoFisherScientific, Waltham, MA, USA).

#### 2.3.6. Octenisept^®^ Release Profile

The spectrophotometric UV detection (UV-Vis Evolution 201, ThermoFisherScientific, Waltham, MA, USA) determined the release profile of OCT components from the hydrogel specimens to PBS (pH of 7.4) and water. Two different calibration curves of OCT in water and PBS were acquired by measuring absorbance at a wavelength of 269 nm, using Octenisept^®^ standard solution. From the above stock, different aliquots were taken separately and diluted to obtain a concentration in the range of 4–130 μg/mL.

To determine the OCT kinetics, the hydrogel specimens were first washed alternately in ultrapure water and ethanol and then kept in 10 mL of the OCT standard solution for 5 days. Afterward, the discs were weighed, dried in a vacuum oven at a temperature of 50 °C for 3 days, and then weighed again. The samples were immersed in the pre-warmed PBS and water at 20 and 37 °C. At various time intervals (5, 15, 30, 60, 120, 180, 240, 300, 360, 1440 min), small volumes (0.2 mL) of the solution were collected and added to vials containing 1.8 mL of water and PBS for spectrophotometric measurements of the OCT concentration. The taken volumes (0.2 mL) were replaced by a new portion of water/PBS. The results were normalized to total cumulative release.

#### 2.3.7. Inhibition of Microorganisms’ Growth Due to the Release of Octenidine Dihydrochloride

The strains applied in this analysis were *S. aureus* ATCC 6538 (ATCC, Manassas, VA, USA), *P. aeruginosa* ATCC 15442, and *Candida albicans* ATCC 10231. The loop of microbial culture grown on Columbia, McConkey, or Saboraoud agar plate, respectively (BioMaxima, Lublin, Poland) was transferred to liquid Tryptone Soya Broth (TSB, BTL, Łódź, Poland) and incubated at 37 °C for 24 h. Next, the microbial culture was diluted in 0.9% NaCl (POCH, Gliwice, Poland) to 0.5McFarland using (Densi-La-Meter II, Erba, Brno, Czech Republic). Then, the microbial culture was transferred into the Muller-Hinton Agar plate (BioCorp, Warszawa, Poland) and swabbed evenly throughout the plate surface. Subsequently, the samples chemisorbed with antiseptics were placed onto the plate. The whole setting was transferred to 37 °C and incubated for 24 h. After incubation, zones of microbial growth inhibition were counted with a ruler. The control setting applied for this experiment were samples that were not chemisorbed with OCT. The procedures performed for this type of sample were performed in the same manner as for OCT-chemisorbed samples. These analyses was performed in triplicates.

#### 2.3.8. The Ability of *Staphylococcus aureus* and *Pseudomonas aeruginosa* to Colonize the Hydrogel

The strains applied in this analysis were *S. aureus* ATCC 6538 (ATCC, Manassas, VA, USA) and *P. aeruginosa* ATCC 15442. The loop of staphylococcal or pseudomonal culture grown on Columbia or McConkey agar plate respectively (BioMaxima, Lublin, Poland) was transferred to liquid Tryptone Soya Broth (TSB, BTL, Łódź, Poland) and incubated at 37 °C for 24 h. Next, the culture’s optical density of 1 McFarland was established with a densitometer (Densi-La-Meter II, Erba, Brno, Czechia). Subsequently, the suspension was diluted in the Miller-Hinton Broth (M-H, BioMaxima, Lublin, Poland) to reach the density of 1 × 10^5^ cells/mL. Next, 1 mL of microbial suspension was introduced to the well of a 24-well plate containing analyzed samples and incubated for another 24 h. After incubation, the samples were gently rinsed with 0.9% NaCl and transferred to the 1 mL of 0.1% saponin (POCH, Gliwice, Poland) and subjected to vigorous vortex-mixing by Multi-Vortex V-32 (Biosan, Riga, Latvia). After mixing, the cell-containing suspension was serially diluted and cultured on appropriate agar plates (Columbia Agar for *Staphylococcus*, McConkey agar for *Pseudomonas*) and incubated at 37 °C for 24 h. After incubation, the number of colony-forming units (cfu) was calculated. The positive control of microbial growth in this experimental setting was bacterial cellulose carrier, which is known for lack of antimicrobial activity and properties facilitating microbial adhesion, while method usability was assessed by application of BC carrier chemisorbed with OCT. The process of BC carrier manufacturing and chemisorption was performed as we described in our earlier work [44]. The subsequent procedures were performed for the hydrogel being the subject of this study. All analyses were performed in triplicates.

#### 2.3.9. Cytotoxicity Tests

The protocol for the determination of the hydrogel’s cytotoxicity was based on the previous study by Junka’s group [45,46]. The normative neutral red (NR) cytotoxicity assay was performed in fibroblast (L929, ATCC, Manassas, VA, USA) cell cultures treated with extracts from hydrogel samples prepared according to the ISO 10993 norm: Biological evaluation of medical devices; Part 5: Tests for in vitro cytotoxicity; Part 12: Biological evaluation of medical devices, sample preparation, and reference materials (ISO 10993-5:2009 and ISO/IEC 17025:2005). Samples were introduced and incubated in a cell line sterile medium in 5% CO_2_ at 37 °C with shaking at 500 rpm for 24 h (Schuttler Microplate Shaker, MTS-4, IKA, Königswinter, Germany). After incubation, they were removed, and crude extracts were spin-centrifuged. The resulted supernatants (extracts) were introduced to the cell cultures and subjected to incubation for 24 h in 5% CO_2_ at 37 °C. Subsequently, the medium was removed and 100 μL of NR solution (40 μg/mL; Sigma-Aldrich, Saint Louis, MO, USA) was introduced to each well of the plate covered with cell lines. Cells were incubated with NR for 2 h at 37 °C. After incubation, the dye was removed while wells were rinsed with Phosphate Buffer Saline (Biowest, Nuaillé, France), and left to dry at room temperature. 150 μL of de-stain solution (50% ethanol, 49% deionized water, 1% glacial acetic acid (*v*/*v*); POCH, Poland) was introduced to each well. The plates were vigorously shaken in a microtiter plate shaker (Plate Shaker-Thermostat PST-60HL-4, Biosan, Riga, Latvia) for 30 min until NR was extracted from the cells. Next, the NR absorbance value was measured with a microplate spectrometric reader (Multi-scan GO, Thermo Fisher Scientific, Waltham, MA, USA) at a 490 nm wavelength. The absorbance value of cells not treated with extracts was considered 100% of potential cellular growth (positive control sample). The negative control constituted fibroblasts exposed for 3 min to 70% ethanol (POCH, Gliwice, Poland). This setting reflects low viability, high reduction of cells. All analyses were performed in 6 repeats.

#### 2.3.10. Statistical Analysis

Measurements were repeated at least three times, and the results were expressed as a mean ± standard deviation. Statistical significance was tested with non-parametric Kruskal-Wallis ANOVA test in OriginPro 9.7 software and was declared as significant (*) at *p* ≤ 0.05.

**Figure 1:**
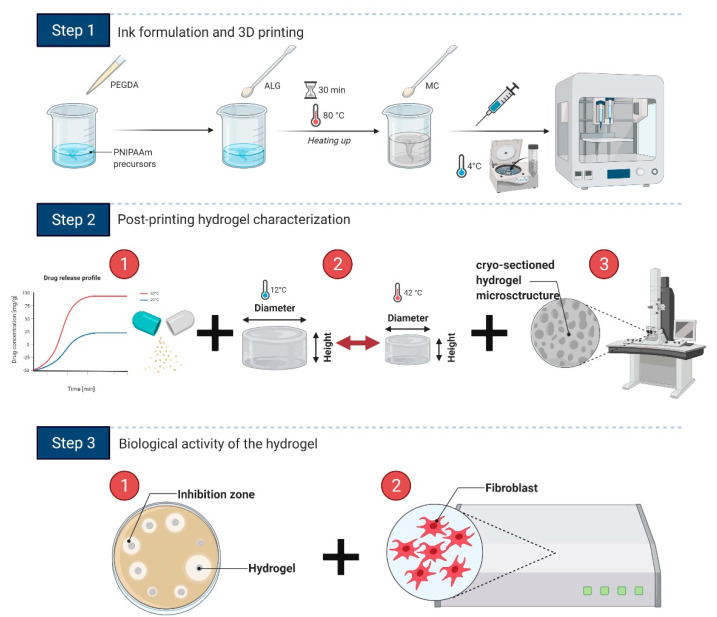
Summary of the procedure: Step 1 demonstrates all steps of the ink formulation and 3D printing including the addition of hydrogel precursors and pre-printing stages such as bubble removal and pouring the ink into a printing syringe; Step 2 presents the main characterization stages that include (**1**) OCT release profile determination, (**2**) swelling tests, and (**3**) SEM observations of the hydrogel cross-sectioned structure; in Step 3, the hydrogel is characterized for its antimicrobial activity and biocompatibility. Created using Biorender.com.

Our protocol of the printable ink synthesis for wound healing applications involves three main steps (Figure 1): (1) the design and formulation of the ink to achieve high printability and shape fidelity, (2) the ink and hydrogel characterization (drug delivery rate, temperature-induced swelling, and microstructure) to find the connection between the hydrogel nature and its biological and thermoresponsive properties, (3) the determination of the antimicrobial activity and biocompatibility of the material toward wound healing applications.

## 3. Results

### 3.1. Characterization of the Ink and Hydrogel

The rheological properties of our ink—which we investigated to determine its printability and shape fidelity—were tuned by adding MC that served as a thickener rendering the ink shear-thinning. The final material contains ALG:MC in a ratio of 1:1 as described in Section 2, Materials and Methods. The hydrogel precursor exhibited a pronounced thixotropic behavior and the ability to maintain a pre-designed shape after deposition.

**Figure 2:**
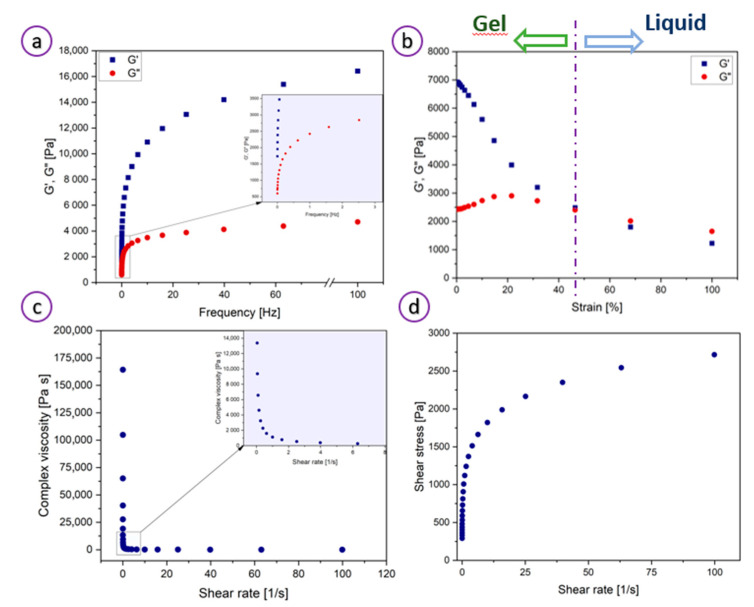
Rheological properties of the hydrogel ink: (**a**) Frequency and (**b**) strain (at a frequency of 1 Hz) dependence of storage and loss moduli. Inset in (**a**) shows the magnified plot for the frequency of 0–10 Hz; (**c**) Complex viscosity of the hydrogel precursor. Inset shows the magnified plot for the shear rate of 0–5 1/s; (**d**) shear stress vs. shear rate plot.

As demonstrated in Figure 2a, the hydrogel showed a higher storage modulus (G′) than the loss modulus (G″) in the indicated frequency range, confirming the elastic behavior of a typical hydrogel. The elasticity (gel-like behavior) results from the strong electrostatic interactions and hydrogen bonds between MC, water molecules, and other ink constituents. At low temperatures (<room temperature), water molecules surround the hydrophobic MC groups rendering the biopolymer water-soluble [47]. While, polar MC groups exhibit a binding affinity towards water species allowing for the formation of highly viscous hydrogel networks [48]. Because of the highly dynamic and reversible nature of those non-covalent interactions, high shear stress (>1 s^−1^) disrupts the bonding structure and instantly switches the material from gel to liquid.

The critical strain (%) value was obtained from the intersection of the two linear parts of the dynamic strain sweep test plots and corresponded to a value of ~46% (Figure 2b). The G′ and G″ vs. % strain plots showed the linear viscoelastic region for strain lower than 46%. Within this region, storage modulus values were higher than those of the loss modulus, indicating the dominating role of elasticity. A sudden decrease in G’ above a critical strain of 46% to values lower than G″ corresponds to a reversible transition between elastic and viscous behavior. Figure 2c shows that at low shear rates (<1 s^−1^), the viscosity is about 175,000 Pa·s, and the system exhibits gel-like properties, whereas above the shear rate of 1 s^−1^ the viscosity dropped drastically to a value of about 20 Pa·s demonstrating liquid-like behavior. A continuous drop in viscosity with increasing shear rates (0–100 s^−1^) confirms the shear-thinning properties of the ink. This shear-thinning and gel-sol transition behavior under stress allow the hydrogel to be extruded easily at low pressures [37]. Figure 2d presents the shear stress in the function of the shear rate. As can be observed, the shear stress increases significantly with the shear rate in the range of 0–20 1/s. At the higher shear rate, the shear stress saturates, indicating that the hydrogel exhibits shear-thinning behavior.

**Figure 3:**
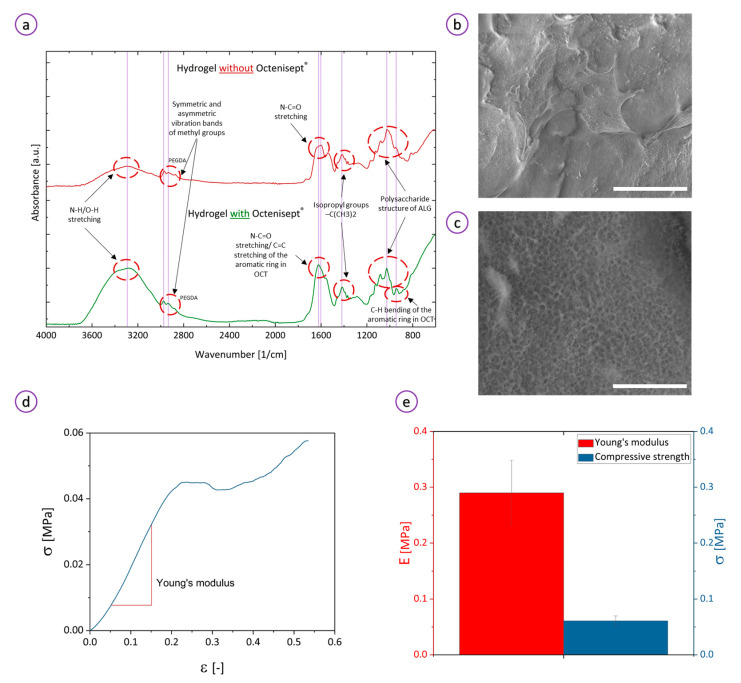
Hydrogel characterization: (**a**) ATR-FTIR spectrum of the 3D-printed sample with (top) and without (bottom) OCT; (**b**,**c**) cryo-SEM images of the cross-sectional microstructure of the specimens. Bars correspond to 40 and 5 μm, respectively; (**d**) stress–strain curve of the hydrogel; (**e**) Young’s modulus and compression strength of the material.

ATR-FTIR analysis identified characteristic groups of the hydrogel components to confirm their presence within the structure. The peaks in the spectrum of thermoresponsive polymers may be shifted towards different positions. These shifts depend on the temperature during measurements because of the molecular changes of the polymer across the LCST transition. Therefore, the spectrum should be analyzed taking into account those temperature-induced differences. We conducted ATR analysis below the lower critical solution temperature (LCST) of PNIPAAm. In the spectrum shown in Figure 3a, a broad band at ~3300 cm^−1^ may correspond to either O-H or amide stretching vibration. The O-H band originates from water molecules entrapped within the matrix and side groups in the polymeric backbone. Peaks at around 2900 cm^−1^ are attributed to symmetric and asymmetric vibration bands of methyl groups of PNIPAAm, ALG, and MC. A peak at 2875 cm^−1^ was associated with the C-H stretching vibration of the carbonyl of the acrylate group, thereby confirming crosslinking of PNIPAAm by PEGDA [6]. The two sharp absorption bands at 1540 cm^−1^ and 1603 cm^−1^ are ascribed to the N–H bending and the N–C=O stretching vibrations in PNIPAAm, respectively [49], whereas a peak at 1388 cm^−1^ was assigned to isopropyl groups of PNIPAAm [50]. Alginate-associated stretching bands (C–O, C–C, C–O–C) characteristic of polysaccharide structure were identified at 1318, 1126, and 1025 cm^−1^ indicating successful incorporation of ALG into the hydrogel. ATR-FTIR analysis of the hydrogel loaded with Octenisept^®^ confirmed the presence of octenidine dichloride and 2-phenoxyethanol. Peaks at ~1630 and 920 cm^−1^ correspond to the C=C stretching and C–H bending vibrations of the aromatic ring, respectively, thereby confirming that Octenisept^®^ has been successfully incorporated into the hydrogel.

Scanning electron microscope (SEM) cryo-images (Figure 3b) showed a smooth surface of the hydrogel. When magnified (Figure 3c), the structure revealed microporous sponge-like surface architecture with a homogenous pore size distribution. A highly regular micropore system arose from the dual crosslinking of the hydrogel via chemical (UV curing) and ionic (Ca^2+^-crosslinking) bonds. Microporous hydrogels may not only help in exchanging the gases and wound fluids and transporting bioactive agents but also constitute a barrier protecting the wound from microbial infection [51]. When juxtaposing IR and SEM results, we may conclude that the hydrogel precursors were uniformly admixed together creating the thermoresponsive hydrogel with a/the homogenous and highly microporous three-dimensional network.

Compressive experimental results show that the hydrogel is soft and can be compressed to a strain of 20% without fracture (Figure 3d). The hydrogel exhibited a compressive strength of 61 ± 9 kPa and an elastic modulus of 0.29 ± 0.06 MPa (Figure 3e). A relatively low compressive strength may result from the nature of the hydrogel which contains physically cross-linked alginate inter-penetrated with chemically-cured PNIPAAm. In this system, MC acts as a non-crosslinked filler and may destabilize the hydrogel’s network.

**Figure 4:**
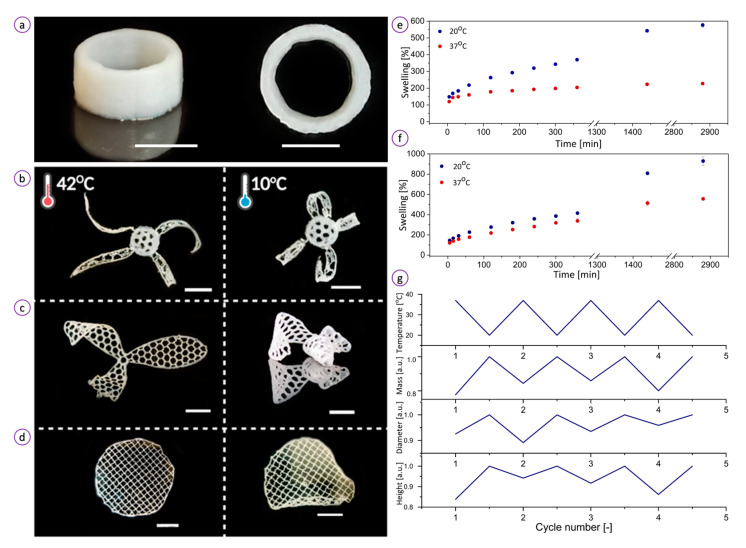
Three-dimensional (3D) printability and temperature-induced behavior of the hydrogel: (**a**) 3D-printed thermoresponsive tube demonstrates high printability of the ink. The combination of 3D printing and ink allows for the fabrication of hollow tubular structures, whose diameter alters in response to temperature change; (**b**) 3D-printed flower-like object with thermoresponsive petals and non-active core; (**c**) 3D-printed thermoresponsive hydrogel propeller; (4) 3D-printed thermoresponsive hydrogel disc. Figure (**b**–**d**) demonstrate that objects printed from the ink can be programmed to exhibit different actuation behaviors at different temperatures (42 and 10 °C). Bars correspond to 1 cm. The hydrogel swelling rates at temperatures 20 and 37 °C in water (**e**) and PBS (**f**); (**g**) Temperature-activated swelling (20 °C) and de-swelling (37 °C) cycles of the sample measured as a change of specimen height, diameter, and mass. The values are normalized to those at a temperature of 37 °C.

To illustrate the printability of the ink, various 3D objects such as a tube (Figure 4a), a flower-like structure with thermoresponsive petals and non-active core (Figure 4b), a propeller (Figure 4c), and a flat disc (Figure 4d) were fabricated by direct printing. To test the preservation of shape fidelity, the macroscopic tubular construct was fabricated as shown in Figure 4a. The tube exhibited expansion-contraction reversibly induced by thermal changes. We found that the tubular sample increased by 10.7% of its original height and original diameter. The results indicate that with the use of our PNIPAAm-based ink, we can create vessel-like geometries that undergo contraction and expansion in response to temperature. Following the previous results [37], we evaluated the shape morphing properties of the hydrogel by visual analysis of the temperature-stimulated shape change of the 3D-printed objects. Upon immersion in cold water (10 °C), the objects tended to fold as a consequence of the in-plane stress generated because of the swelling. When transferred to water maintained at 42 °C, the structures shrank and refolded as a result of a contractive stress release. In Figure 4b, this process is demonstrated as a flower reversible blossoming with a temperature change.

### 3.2. Temperature-Triggered Behavior of the Hydrogel

One of the major concerns regarding hydrogels for wound dressings is their water absorption capacity. The intrinsic microstructure and the physicochemical properties of hydrogels affect their water absorption efficacy and in turn the swelling ratios. PNIPAAm and PNIPAAm-containing hydrogels undergo temperature-responsive coil–globule or volume phase transitions that are associated with swelling/shrinkage behaviors. The swelling of the PNIPAAm-based hydrogel involves the solvation of functional groups in the polymer backbone that dominate over the polymer-polymer interactions [52]. The swelling kinetics of the hydrogel specimens placed in water at temperatures of 20 and 37 °C are presented in Figure 4e. When immersed in water at a lower temperature, a dry hydrogel specimen began to swell until it achieved 577 ± 12% of its initial mass after 48 h of incubation. The swelling ratio at a temperature of 37 °C reached only ~50% (228 ± 7%) of the value at 20 °C during 48 h. A similar trend was observed for the sample incubated in PBS (Figure 4f). At 20 °C, the equilibrium swelling ratio was 930 ± 42%. However, at the higher temperature, the hydrogel showed an almost twice lower swelling ratio of 556 ± 14%. The swelling ratio in PBS at 37 °C reached almost the same value as the sample kept at 20 °C in water. This means that the hydrogel undergoes environment-dependent swelling behavior. All curves, however, showed a gradual increase in the swelling ratio during incubation. The results suggest that the water absorption capacity of the hydrogel can be tuned by adjusting its temperature.

Figure 4g demonstrates that the hydrogel can reversibly switch between swollen and deswollen states in four 120 min consecutive swelling–contraction cycles. The hydrogel mass decreased to an average ~82 ± 3% of its initial value at 20 °C within 120 min when placed in water at 37 °C, resulting in the reduction of height and diameter by ~8 and 11%, respectively. A slight change in dimensions upon transfer from cold to hot water suggests that the hydrogel can preserve its geometrical size while retaining a high amount of water. This property of our hydrogel may be beneficial for personalized wound care to produce patches with precisely controlled geometry that matches wound shape.

**Figure 5:**
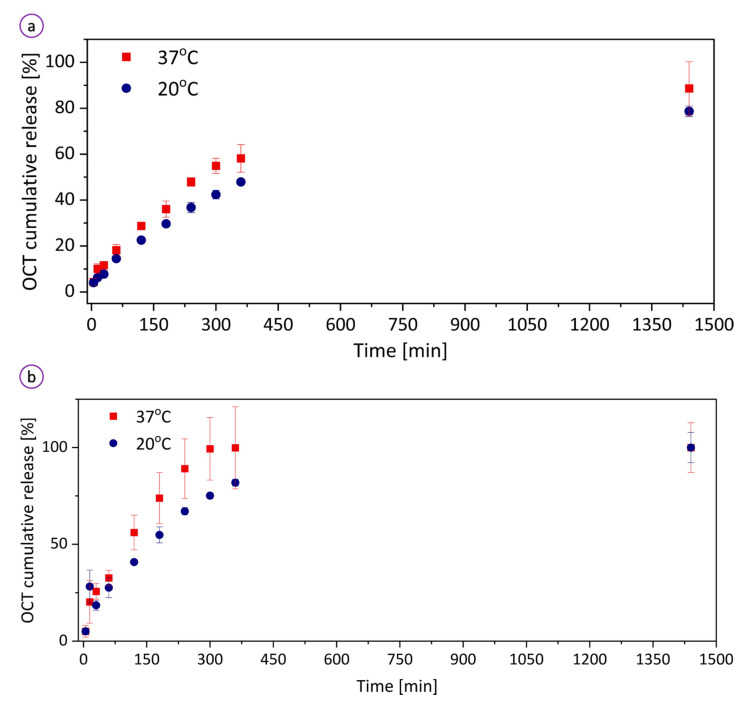
OCT release profiles from the hydrogel to (**a**) water and (**b**) PBS (pH of 7.4) at temperatures of 20 and 37 °C.

To assess the drug release kinetics, we examined the OCT release profile in vitro of the hydrogel at two different temperatures (20 and 37 °C) to water and PBS solution (pH of 7.4) (Figure 5). The hydrogel demonstrated a temperature-dependent release of OCT. At temperatures below the LCST of PNIPAAm, the hydrogel showed a distinctly prolonged release profile. Below the LCST, the polymer backbone is highly hydrated and remains coiled, while a drug passively diffuses through the polymer matrix. At higher temperatures above the LCST, the polymer chain collapses into a compact globule state causing the drug to be “squeezed out” from the hydrogel [53]. It has been reported that the PNIPAAm composites laden with a drug by the physical loading strategy release faster at temperatures above LCST [54,55]. Our study confirmed this observation as OCT was released more rapidly at 37 °C than that at 20 °C. At higher temperatures (above LCST), the molecular network is contracted, which fosters rapid water desorption and simultaneous drug release. Below the LCST, the OCT diffuses in the highly swollen hydrogel network overpassing hydrophilic interactions with the backbone. When immersed in water, our hydrogel steadily delivers OCT until it reached the cumulative release of 91.3 ± 11.7 and 77.2 ± 2.3% at 37 and 20 °C, respectively. This means that the 24 h-incubation was insufficient to achieve 100% efficiency in the delivery of OCT. In PBS at 37 °C, the sample released ~100% of the encapsulated OCT within 5 h, whereas at 20 °C OCT was completely (99.2 ± 7.8%) delivered within 24 h. The difference in the rates for water and PBS results from the partial degradation of the hydrogel in the buffer solution, which is slower in water. The composite contains physically cross-linked alginate which discharges calcium ion in exchange for sodium ion in the PBS buffer leading to the degraded hydrogel architecture. At temperatures above the LCST, the polymer contraction causes pushing the drug out of the network, hence OCT is completely released in less than 24 h. At 20 °C, the hydrogel absorbs a large amount of water, while the drug diffuse through the water-filled pores. Relaxation of molecular chains due to swelling helps the drug to diffuse out of the swollen polymer. The results showed that the release of OCT from the hydrogel can be controlled by temperature and type of external environment.

### 3.3. Biological Activity of the Hydrogel

Gram-positive *Staphylococcus aureus* and Gram-negative *Pseudomonas aeruginosa* are considered bacteria of high invasive potential and they are commonly isolated from infected chronic wounds of various etiology [56]. Wounds are also highly susceptible to infection by a yeast-like fungus referred to as the *Candida albicans* [57]. Therefore, in this study *S. aureus* ATCC 6538, *P. aeruginosa* ATCC 15442, and *C. albicans* ATCC 10231 were selected to evaluate the antibacterial efficacy of the hydrogel.

**Figure 6:**
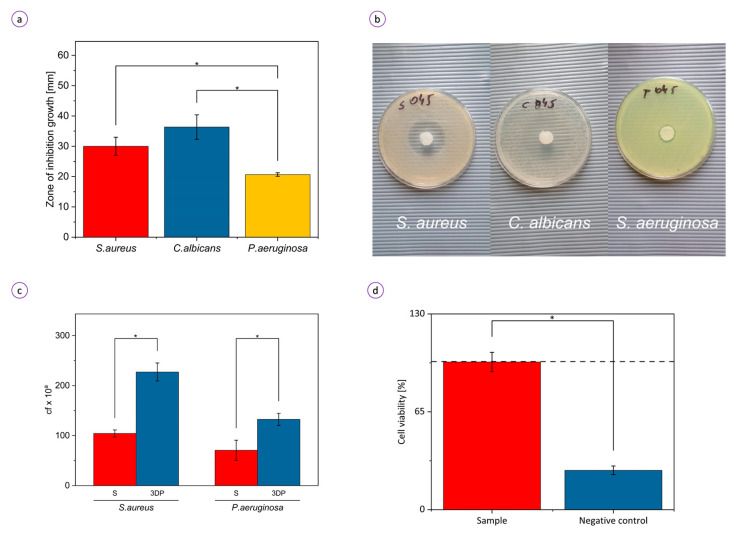
Biological activity of the hydrogel: (**a**) average diameter of inhibition zones of *S. aureus, C. albicans,* and *P. aeruginosa* and (**b**) corresponding photos of the dishes; (**c**) ability of *S. aureus* and *P. aeruginosa* to colonize the molded (S) and 3D-printed (3DP) hydrogel samples. An exponent “a” corresponds to 9 and 6 for *S. aureus* and *P. aeruginosa*, respectively; “cf” means colony-forming; (**d**) viability of fibroblasts exposed to medium conditioned with extracts from the analyzed sample and ethanol (negative control). Absorbance at 490 nm measured for unexposed fibroblasts (C) was considered 100% (black dashed line); * *p* < 0.05.

The OCT-containing PNIPAAm/ALG hydrogel effectively inhibited microbial growth. The growth inhibition haloes were observed around the hydrogel discs laden with Octenisept^®^ (Figure 6a,b). The gradient of the released antiseptic prevents the growth of microbes creating an “inhibition zone” around the specimen. The smallest inhibition zone diameter was observed for *P. aeruginosa* (20.7 ± 0.5 mm)*,* while there was no statistically significant difference in sensitivity of *S. aureus* (30.0 ± 2.5 mm) and *C. albicians* (36.3 ± 3.3 mm) to the OCT-releasing hydrogel. The provided control setting (hydrogel without OCT) revealed that hydrogels non-chemisorbed with antiseptic possess no intrinsic antimicrobial activity (Figure S1 in Supplementary Material). The results suggested that drug molecules released from the hydrogel retain their antimicrobial capability. High antimicrobial activity results from the incorporation of OCT within the material matrix and the three-dimensional microstructure with homogenous microporosity. The observed differences in halo zone sizes may be the result of different inter-species tolerance levels against OCT. These findings indicate the promising prospect of hydrogel as a highly potent antibacterial material for wound dressing applications.

Colonization tests were conducted against *S. aureus* and *P. aeruginosa* to determine the level of bacterial adhesion on the surface of the molded and 3D-printed hydrogel samples without OCT. The control setting in which adhesion-facilitating BC carrier was applied, showing that *S. aureus*, *P. aeruginosa,* and *C. albicans* were able to colonize BC and reach the number of colony-forming units of 10^10^, 10^10^, and 10^8^/sample, respectively. The analysis of the ability of pathogenic microorganisms to adhere to the hydrogel is presented in Figure 6c. There were 46 and 53% fewer *P. aeruginosa* and *S. aureus* adhered to the molded hydrogel than to the 3D-printed one, respectively. 3D-printed porous hydrogel scaffold favored the attachment of microorganisms, which implies bacteria adhere and colonize the porous surface preferentially.

To determine the biocompatibility of the hydrogel, its toxic effect towards fibroblasts (L929) was tested with a standard (NR) cytotoxicity assay. Fibroblasts (L929) were selected as the most appropriate cell line for wound healing studies [58]. As shown in Figure 6d, no significant cytotoxicity in vitro was observed in response to the hydrogel extracts. More than 98% as compared to the control (black dashed line) of the cells remained viable. The hydrogel was found to remain stable in cell culture media for 24 h at 37 °C, indicating that the hydrogel can be applied for antimicrobial biomedical purposes without causing long-term effects on biological growth. The low cytotoxicity of the PNIPAM-based hydrogel makes it an interesting material with clinical potential in tissue engineering, e.g., for the fabrication of artificial temperature-actuated vessels.

## 4. Discussion

PNIPAAm and its copolymers have been extensively investigated over the last decades [59,60], but adapting PNIPAAm for wound healing applications by creating an interpenetrating network of dually-crosslinked thermoresponsive polymer with alginate and MC pursues a unique path. We have developed a novel 3D-printable Octenisept^®^—containing thermoresponsive hydrogel that has great antibacterial and antifungal activities. A fundamental concern for the clinical biomedical application of hydrogel is its bio-compatibility [61]. Non-cytotoxicity is one of the most important criteria determining the feasibility of hydrogels as wound healing dressings. Although we used components that have been proved to be biocompatible, their combination in a composite material has not been tested for cytotoxicity [62,63,64,65]. Moreover, calcium alginate dressings have shown positive effects on wound healing by providing a moist wound environment [63]. We exploited PNIPAAm thermoresponsivity, alginate and PEGDA biocompatibility, and MC shear-thinning properties to engineer inks for direct printing of multifunctional thermo-triggered hydrogels with temperature-dependent swelling, shape morphing, and drug release behavior. The hydrogel exhibited detectable antimicrobial activity against common wound pathogens (*Staphylococcus aureus, Candida albicans,* and *Pseudomonas aeruginosa*) while demonstrating non-cytotoxicity towards the fibroblast (wound repairing cells) line (L929).

The good printability of the inks confirms their feasibility to design and fabricate wound closures that can perfectly match the unshaped wound towards personalized medicine. The current trend toward customized healthcare means that traditional dressings, e.g., cotton and wool bandages and gauzes that have no active function in the healing process will be replaced by a new generation of advanced and individualized wound closures functionalized with different therapeutic ingredients to be delivered to wound sites. In this study, we follow that direction and propose multifunctional printable hydrogels laden with the antiseptic that can be used for on-demand purposes with the assistance of 3D direct printing. Another advantage worth mentioning is that by loading the hydrogel with the non-specific antiseptic drug, we have developed a drug delivery platform that exhibits high efficiency in killing both Gram-positive and Gram-negative bacteria, and fungi colonizing a wound. One should notice that the microporous structure of the hydrogel may potentially prevent microorganisms from penetrating the wound, but on the other hand, materials of developed structure may also facilitate micro-organisms adhesion on them. Therefore, chemisorption of hydrogel with OCT antiseptic may serve not only as a weapon against microorganisms already infecting the wound but also as a shield protecting hydrogel itself from contamination. Because of the hydrogel’s thermoresponsivity, the OCT release profile can be tuned by changing a medium temperature.

We proved the hydrogel’s multifunctionality and demonstrated a number of its applications including wound dressings, drug delivery systems, vessel and stents. Further investigation is needed to explore the effect of hydrogel treatment on the promotion of wound healing in the experiments in vivo. This could be the basis for further research and development of clinically used thermoresponsive hydrogels with a drug delivery function for wound care products. In perspective, the research on 3D-printed shape memorable tubes should be expanded to evaluate their potential for mimicking blood vessels. Although the shape morphing behavior was confirmed, the exact mechanism of temperature-induced deformation has not been quantitatively determined.

## 5. Conclusions

We have developed a new biomaterial ink for direct printing and molding of temperature-triggered biocompatible hydrogel constructs. The ink was highly viscous and exhibited shear-thinning behavior, which translated into its good printability and shape fidelity. The hydrogel characterization results indicated that the hydrogel components were merged uniformly forming a homogenous and microporous intrinsic structure. The hydrogel was further laden with Octenisept^®^ to render the hydrogel antimicrobial and expand the applications to healing dressings. Because of the introduction of temperature-stimulated polymer, the material was able to sense locally the temperature and regulate the swelling ratio and drug diffusion. The 3D-printed samples showed temperature-induced shape morphing behavior, and hence the potential application of a new generation of wound dressings.

## Data Availability

All data generated or analyzed during this study are present in the main text. Additional data are available on request from the corresponding author.

## Supplementary Materials

The following are available online at https://www.mdpi.com/article/10.3390/bioengineering8060079/s1, Figure S1: Lack of intrinsic antimicrobial activity of analyzed samples against (A) *S. aureus*, (B) *P. aeruginosa* and (C) *C. albicans*. Pictures A1, B1, C1 are 4 × magnifications of pictures A, B, C respectively, focused on the sample and sample surroundings.
