# Supplementary material for: 3D Printing of Thermoresponsive Hydrogel Laden with an Antimicrobial Agent towards Wound Healing Applications
Source: Bioengineering (Basel). 2021 Jun 8;8(6):79. doi: 10.3390/bioengineering8060079 (PMC8227034; doi:10.3390/bioengineering8060079)
Supplement: Supplementary file 1 [file bioengineering-08-00079-s001.zip › bioengineering-1196297-SI.pdf]

Supplementary Material

3D printing of thermoresponsive hydrogel laden with an antimicrobial agent toward wound healing applications

Martyna Nizioł[1], Justyna Paleczny[2], Adam Junka[2], Amin Shavandi[3], Anna Dawiec-Liśniewska[4], and Daria Podstawczyk[1,*]

[1] Department of Process Engineering and Technology of Polymer and Carbon Materials, Faculty of Chemistry, Wroclaw University of Science and Technology, Norwida 4/6, Wroclaw, 50-373 Poland; martyna.niziol@pwr.edu.pl, daria.podstawczyk@pwr.edu.pl
[2] Department of Pharmaceutical Microbiology and Parasitology, Wroclaw Medical University, 50-556, Wroclaw, Poland; adam.junka@umed.wroc.pl, justyna.paleczny@student.umed.wroc.pl
[3] BioMatter Research Unit - Biomass and Biomaterials (3BIO-BioMatter), Université Libre de Bruxelles, Brussels, Belgium, amin.shavandi@ulb.be
[4] Department of Advanced Material Technology, Faculty of Chemistry, Wroclaw University of Science and Technology, Wroclaw 50-372, ul. M. Smoluchowskiego 25, Poland, anna.dawiec@pwr.edu.pl
* Correspondence: daria.podstawczyk@pwr.edu.pl; Tel.: +48713203437

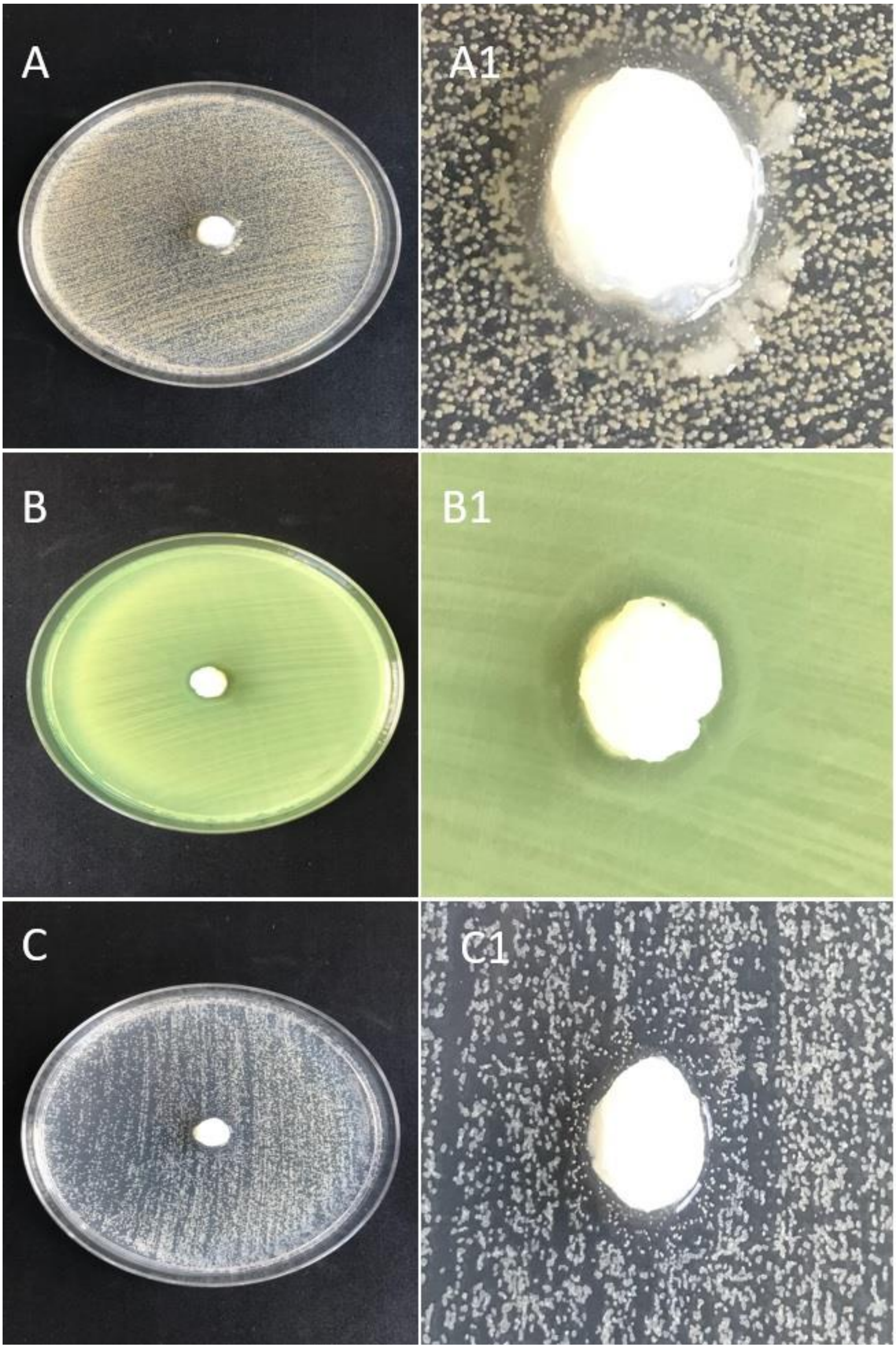
A
A1
B
B1
C
C1

Figure S1. Lack of intrinsic antimicrobial activity of analyzed samples against A) *S.aureus*, B) *P.aeruginosa* and C) *C.albicans*. Pictures A1, B1, C1 are 4x magnifications of pictures A, B, C respectively, focused on the sample and sample surroundings.
